# INDIVIDUAL PREDICTORS OF VOLUNTEERING IN OLDER ADULTS USING STRUCTURAL EQUATION MODELING

**DOI:** 10.1093/geroni/igz038.582

**Published:** 2019-11-08

**Authors:** Zolana J Baumel, Christopher M Kelly

**Affiliations:** University of Nebraska - Omaha, Omaha, Nebraska, United States

## Abstract

Why do some older Americans volunteer while others do not? Personality theory is used to explain volunteering behavior. This study used the Big 5 personality domains. Volunteering is also explained by resource theory, which includes personal, social, and cultural capital. Personal capital was measured by age, sex, race, marital status, health, and education (a proxy for socioeconomic status). Social capital was measured by frequency of email/write interactions and interactions with friends. Cultural capital was measured as church attendance. The purpose was to measure the extent to which social and cultural capital mediates between personality and personal capital, and volunteering outcomes. Participants were the 2010 Core Health and Retirement Study who also completed the leave-behind self-administered questionnaire (N=4,299). Structural equation modeling (SEM) tested personality and resource theory in one model. Exploratory factor analysis, followed by confirmatory factor analysis supported development of the final SEM. Extraversion directly predicted volunteering. Age was inversely correlated with volunteering. Health and education directly predicted volunteering. Church attendance and interactions with friends directly predicted volunteering. Conscientiousness predicted church attendance. Open to new experience was inversely related to church attendance and directly predicted frequency of email/write interactions. Extraversion predicted interactions with friends. Measuring direct, indirect, and total effects of predictors on volunteering showed that personality influences volunteering both directly and indirectly. Future applied research could test whether extraversion, which directly influences interactions with friends, would directly predict recruiting outcomes. It could also test whether open to new experience, which directly influences email/write interactions, would directly predict retention outcomes.

